# Preconditioned and Genetically Modified Stem Cells for Myocardial Infarction Treatment

**DOI:** 10.3390/ijms21197301

**Published:** 2020-10-02

**Authors:** Kamila Raziyeva, Aiganym Smagulova, Yevgeniy Kim, Saltanat Smagul, Ayan Nurkesh, Arman Saparov

**Affiliations:** Department of Medicine, School of Medicine, Nazarbayev University, Nur-Sultan 010000, Kazakhstan; kamila.raziyeva@nu.edu.kz (K.R.); Aiganym.Smagulova@nu.edu.kz (A.S.); Yevgeniy.Kim@nu.edu.kz (Y.K.); ssmagul@nu.edu.kz (S.S.); ayan.nurkesh@nu.edu.kz (A.N.)

**Keywords:** myocardial infarction, stem cell therapy, stem cell preconditioning, genetic modification, cell reprogramming, stem cell differentiation

## Abstract

Ischemic heart disease and myocardial infarction remain leading causes of mortality worldwide. Existing myocardial infarction treatments are incapable of fully repairing and regenerating the infarcted myocardium. Stem cell transplantation therapy has demonstrated promising results in improving heart function following myocardial infarction. However, poor cell survival and low engraftment at the harsh and hostile environment at the site of infarction limit the regeneration potential of stem cells. Preconditioning with various physical and chemical factors, as well as genetic modification and cellular reprogramming, are strategies that could potentially optimize stem cell transplantation therapy for clinical application. In this review, we discuss the most up-to-date findings related to utilizing preconditioned stem cells for myocardial infarction treatment, focusing mainly on preconditioning with hypoxia, growth factors, drugs, and biological agents. Furthermore, genetic manipulations on stem cells, such as the overexpression of specific proteins, regulation of microRNAs, and cellular reprogramming to improve their efficiency in myocardial infarction treatment, are discussed as well.

## 1. Introduction

Cardiovascular diseases (CVD) remain one of the most serious contemporary health issues, accounting for substantial morbidity and mortality throughout the world. According to the Global Burden of Diseases data, in 2017 alone, there were about 17.8 million deaths globally due to CVD [1]. The Heart Disease and Stroke Statistics-2020 Update states that the number of deaths attributed to CVD has increased by 21.1% over the period of 2007–2017. It was estimated that in 2017, 485.6 million individuals had CVD, which corresponds to a 28.5% increase over 10 years [2]. The economic burden of CVD is enormous as well. For instance, in the US alone, the overall spending on CVD rose by 147% between 1996 and 2015, reaching 318 billion USD [3]. Importantly, based on projections, this number is expected to rise to $1.1 trillion USD in 2035 [4]. Among CVD, ischemic heart disease with myocardial infarction (MI) as an important constituent is the number one cause of mortality worldwide [5,6]. In 2017 alone, about 8.93 million people across the globe died from ischemic heart disease [1]. MI can lead to heart failure that accounts for an approximate 50% death rate within five years after diagnosis [7]. MI leads to the death of cardiomyocytes, and because the regenerative capacity of the heart is limited, this loss is mostly irreversible [8,9]. Conventional treatment for MI is coronary reperfusion therapy, such as percutaneous coronary intervention, fibrinolytic therapy or coronary artery bypass grafting [10]. Unfortunately, these therapeutic strategies cannot improve the repair and regeneration of the infarcted myocardium; instead, they only attempt to prevent further damage of the heart muscle and avoid subsequent ischemic episodes [8]. Heart transplantation is an available option to replace the infarcted heart. However, this strategy has obvious limitations such as shortage of donor hearts, high cost, complicated surgery, and the need for immunosuppression after transplantation, among others [11]. The limitations of existing MI therapies necessitate the search for alternative treatment methods that are able to replenish the lost myocardial tissue and are also safe, cost-effective, and implementable in clinical practice. Therapy with stem cells seems to be a promising alternative to the available treatments of MI [12].

A wide range of studies have been performed on the efficacy of stem cell therapy for cardiac repair over the last two decades [13,14]. Stem cells are unspecialized cells that possess unique abilities to renew themselves and to differentiate into other cell types [15]. Different types of stem cells such as hematopoietic stem cells, endothelial progenitor cells, mesenchymal stromal/stem cells (MSCs), skeletal myoblast-derived stem cells, cardiac stem cells, embryonic stem cells (ESCs), and induced pluripotent stem cells (iPSCs) have been studied in these trials. Their positive effects are mediated through three main mechanisms: direct differentiation into cardiomyocytes, differentiation into vascular cells, and paracrine signaling [16]. MSCs are multipotent adult stem cells that have shown promising results for cardiac tissue repair [17,18]. According to criteria from the International Society for Cellular Therapy, MSCs can be defined as self-renewable, multipotent stem cells that can differentiate into osteoblasts, chondroblasts, and adipocytes as well as possess a specific profile of surface markers by expressing CD73, CD90, and CD105, and lacking CD14, CD34, CD45, and HLA-DR [19]. However, it was shown that cells with the aforementioned characteristics show a profound heterogeneity in terms of proliferation rate, aging, degree of stemness, and cellular fate [20]. This largely depends on the source of MSCs, which can be obtained either from fetal or adult tissues and organs. MSCs of fetal origin are obtained from placenta, umbilical cord, amniotic fluid, chorionic villi, and Wharton’s jelly [21,22]. Adult MSCs can be isolated from multiple tissues and organs such as bone marrow, fat, dental pulp, skin, lung, liver, and many others [21,22]. It was shown that fetal MSCs have a higher proliferation rate and senesce at later passages in vitro, whereas adult bone marrow-derived MSCs have greater stemness [20]. MSCs obtained from different tissues also vary in their differentiation fate. For instance, umbilical cord MSCs differentiate preferentially toward adipocytes, whereas bone marrow and adipocyte-derived MSCs mainly give rise to osteoblasts [20]. The origin of MSCs is also controversial. One theory states that MSCs are derived from embryonic mesenchymal tissue, which in turn originates from mesoderm. Another hypothesis is that specific neural crest cells give rise to MSCs. Another hypothesis is that specific neural crest cells give rise to MSCs [20]. It is likely that all of these theories are accurate, i.e., MSCs are a population of highly heterogenic cells of different origins. Contrary to original speculation, MSCs mediate their regenerative effects mainly by stimulating and supporting the resident cells rather than replacing them [20,23]. Thus, they contribute to the repair of injured tissues mainly by releasing various trophic and paracrine factors as well as suppressing immune response. By contrast, the differentiation of MSCs to resident cells plays a negligible role in tissue restoration. This holds true for the field of cardiac regeneration as well. Thus, the ability of MSCs to differentiate into cardiomyocytes remains disputable [24]. Despite promising results in preclinical studies, there are several obstacles standing in the way of clinical implementation of stem cell therapy. These include extremely low survival and engraftment, limited differentiation after transplantation, safety concerns such as potential teratogenicity and immune reactions, ethical issues associated with the use of ESCs, and other factors [16,25]. Moreover, in order to promote the clinical application of stem cells, major issues including stem cell source and type, dosage, route and time of administration, engraftment, differentiation, as well as mechanism of action need to be considered [26].

A number of strategies have been utilized in order to address the challenges associated with stem cell therapy. Preconditioning and genetic manipulations are used to optimize stem cell transplantation therapy for MI. Preconditioning refers to the exposure of stem cells to various physical and chemical factors in vitro [27]. Preconditioning with hypoxia, anoxia, heat shock, medications or other chemicals, growth factors (GF), cytokines, as well as genetic modification of stem cells to overexpress specific cell surface proteins, enzymes, or signaling molecules have been successfully tested in preclinical models [28,29]. Multiple studies have shown that preconditioning improves survival and engraftment of the transplanted cells, enhances their paracrine effects, and boosts their capacity for tissue regeneration following MI by diminishing fibrosis and cardiomyocyte loss, upregulating angiogenesis, and preserving cardiac function [30,31,32]. In the present paper, we will review the most recent studies that utilize various preconditioning and genetic modification approaches for the optimization of stem cell transplantation therapy in an experimental model of MI. In the first part of the review, we will focus on preconditioning strategies such as hypoxia, GF, drugs, and biological agents. The second part, in turn, will be dedicated to genetic modification, the overexpression or inhibition of specific microRNAs, cell reprogramming, and stem cell differentiation.

## 2. Stem Cell Preconditioning for MI Treatment

**Figure 1:**
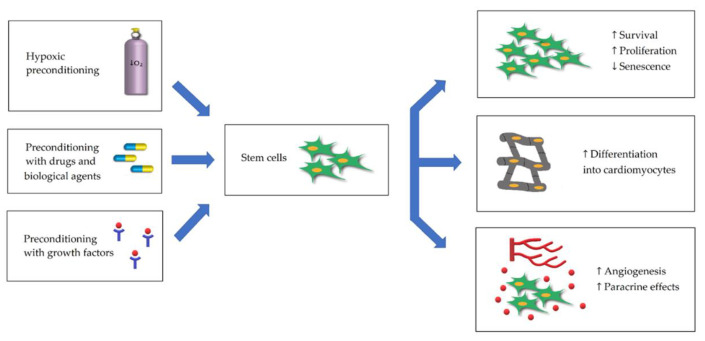
Preconditioning strategies to improve myocardial infarction stem cell therapy. Hypoxic preconditioning, preconditioning with drugs/biological agents and growth factors have been utilized to improve survival and proliferation, enhance differentiation, increase paracrine activity and angiogenesis.

Upon transplantation, the stem cells are confronted with a harsh environment at the MI site, which results in a lower engraftment rate, poor survival, and weak proliferative abilities, therefore limiting the effectiveness of the therapy [33]. The in vitro preconditioning of stem cells aims to prepare the cells for the ischemic environment prior to transplantation and is among the possible solutions that have demonstrated significant enhancement in infarction site recovery [34,35]. The cells can be preconditioned via various methods, including but not limited to pretreatment with hypoxia, drugs, biological agents, and growth factors (Figure 1) [31,36,37].

### 2.1. Hypoxic Preconditioning of Stem Cells

In early 2000, a group of scientists discovered that rat MSCs pre-incubated in low oxygen conditions (5% oxygen) had better bone-forming abilities when implanted into syngeneic host animals [38]. Further investigations found that primary MSCs are naturally highly concentrated in the surface of trabecular bones, where normal oxygen levels vary between 1% and 7%. The self-renewal capacity and lifespan of cells there were much higher, indicating the importance of maintaining low oxygen conditions for stem cells [39]. Moreover, the infarcted area is also strongly hypoxic. Therefore, culturing stem cells in normoxic (≈20% of oxygen) conditions may be a reason for the low engraftment rate [40]. Hypoxic preconditioning (HPC) could advance the efficiency of stem cell therapy. Indeed, in multiple studies, the exposure of stem cells to hypoxic conditions prior to transplantation resulted in improved survival, enhanced proliferation and differentiation, and reduced senescence [41,42,43,44]. The alternation of hypoxic and normal oxygen conditions promotes multipotency and the expression of pro-survival genes and trophic factors in MSCs [31,45].

Stem cells possess antioxidative defense mechanisms, which mediate their survival in the harsh environment [46,47]. Therefore, manipulations with oxygen tension in stem cells prior to transplantation have a beneficial effect on cell survival and their tissue regenerative potential [40,48]. HPC activates the phosphoinositide 3-kinase (PI3K)-Akt signaling pathway and subsequently enhances the production of angiogenic and anti-apoptotic factors, such as hypoxia-inducible factor-1, angiopoietin-1, vascular endothelial growth factor (VEGF) and its receptor, Flk-1, erythropoietin, Bcl-2 and Bcl-xL [40,49]. In addition, HPC facilitates the translocation of DJ-1 from the cytosol to the mitochondria in a complex with Grp75. In the mitochondria, DJ-1 cooperates with subunits of mitochondrial complex I (ND1 and NDUFA4), which protects mitochondrial complex I activity and inhibits reactive oxygen species formation [50]. Long-term hypoxic preconditioning enhanced the cardioprotective properties of bone marrow stem cells (BMSCs) and downregulated the expression of pro-inflammatory and pro-fibrotic cytokines, whereas the expression of anti-inflammatory and angiogenic cytokines was upregulated in MI tissue. Compared to normoxic conditions, hypoxic preconditioned BMSCs showed better clonogenic potential and cell proliferation [51]. In addition, the studies demonstrated the increased viability of BMSCs at the site of infarction, enhanced heart function, and an increase in VEGF and transforming growth factor-β (TGF-β) expression. The latter proteins were associated with collagen deposition in fibroblasts via PI3K/Akt and TGF-β/mothers against decapentaplegic homolog 2 (SMAD2) pathways, and VEGF also promoted vascularization at the MI site [51]. Although preconditioning for 24 h significantly enhanced paracrine secretion and the survival of MSCs in post myocardial tissue through the regulation of autophagy, 48 h preconditioning diminished the expression of VEGF, basic fibroblast growth factor (bFGF or FGF2), hepatocyte growth factor (HGF), and insulin-like growth factor-1 (IGF-1) and enhanced the level of apoptosis and autophagy in tissue [52]. Furthermore, HPC in combination with curcumin significantly reduced the apoptosis of transplanted BMSCs and restored mitochondria function, which is a critical indicator of cell death following MI [53,54].

The first large-scale preclinical study on non-human primates confirmed the improvement of myocardial functions and the significant reduction of infarct size after treatment with hypoxic MSCs. Beneficial effects and the absence of complications suggest the feasibility of using hypoxic MSCs in clinical trials to treat myocardial tissue dysfunctions [55]. Additional studies that used hypoxic MSCs transplantation to non-human primates with MI showed an increase in the number of endothelial and smooth muscle cells as a consequence of angiogenesis. Overall, the hypoxic preconditioning of stem cells, prior to transplantation into the infarcted myocardium, significantly improves cardiac functions via enhanced survival, proliferation, and differentiation, as well as the increased expression of angiogenic and anti-apoptotic factors.

### 2.2. Preconditioning with Growth Factors

Stem cell preconditioning with GF could advance their therapeutic efficiency (Figure 1). This type of preconditioning results in improved cell survival and proliferation, decreased apoptosis, enhanced cellular differentiation, and increased paracrine activity in vitro [56,57]. Stem cells preconditioned with GF and small molecules were successfully tested to treat MI in animal models [30,36]. Guo and colleagues examined the effects of IGF-1 pretreatment on bone marrow MSCs in a rat model of MI [58]. The authors reported that IGF-1-treated MSCs survived better after transplantation in comparison to non-treated cells. In addition, detrimental post-MI sequelae such as cardiomyocyte death, ventricular wall thinning, dilatation, and fibrosis were significantly milder in the case of preconditioned cells. Importantly, the treatment resulted in the decreased production of several pro-inflammatory cytokines, namely tumor necrosis factor-α, interleukin-1β (IL-1β), and IL-6 in the infarction area. One limitation of the IGF-1 pretreatment is that it is not clear whether this GF can promote differentiation of MSCs into cardiomyocytes [59]. On the other hand, HGF is a known regulator of MSCs differentiation into cardiomyocytes [60]. Therefore, Zhang and colleagues attempted to complement the IGF-1 preconditioning of stem cells with HGF pretreatment [59]. MSCs preconditioned with the combination of the two growth factors differentiated into cardiomyocytes both in vitro and in a rabbit model of MI. The pretreatment also attenuated the infarct size, improved cardiac function, and promoted angiogenesis.

bFGF is another molecule that can be utilized to advance the efficiency of stem cells. Ling and colleagues demonstrated that the pretreatment of Sca-1+ cardiac stem cells with bFGF enhanced their targeting to the infarct site as well as improved angiogenesis [61]. The authors claimed that the migration of the preconditioned cardiac stem cells was mediated via the PI3K/Akt pathway, since the inhibition of this pathway diminished the migratory properties of the cells. Overall, the preconditioning of stem cells with GF appears to be an effective strategy to account for the limitations of stem cell transplantation therapy. GF-pretreated stem cells have been widely used for a variety of clinical conditions [31]. Thus, recent studies have shown that stem cell preconditioning can be productively utilized for the treatment of MI.

### 2.3. Preconditioning with Drugs and Biological Agents

Various biological and chemical/pharmacological substances have also been used for stem cell preconditioning. Lee and colleagues reported that the pretreatment of human adipose-derived stem cells (ADSCs) with n-butylidenephthalide (BP), an active compound of Angelica sinensis, promotes M2 macrophage attraction to the injured tissue. Preconditioned cells showed better survival, retention, and differentiation into cardiomyocytes through regulation of the PI3K/Akt/glycogen synthase kinase-3 beta (GSK-3β)-dependent pathway [62]. Later, the authors discovered a novel BP-ADSCs regulatory mechanism in cardiac post-MI tissue. Their study demonstrated that the transplantation of BP-ADSCs into infarcted hearts attenuated myocardial fibrosis through the polarization of macrophages toward a reparative M2 phenotype and positive regulation of the PI3K/signal transducer and activator of transcription 3 (STAT3) pathway [63]. In another study, N-cadherin (Ncad) overexpression in ADSCs resulted in an increased survival of stem cells in an MI mouse model. Additionally, ADSCs-Ncad treatment enhanced heart recovery, promoted angiogenesis and cell proliferation, and reduced fibrosis [64].

Improved cardiac function and attenuated scarring was observed in an MI model treated with Cardiotrophin-1 transduced BMSCs. An in vitro analysis demonstrated that preconditioned cells inhibited cellular apoptosis and promoted cell adhesion [65]. Moreover, the treatment of BMSCs with fibronectin type III domain-containing protein 5 enhanced the engraftment and production of paracrine factors, reduced fibrosis, and recovered cardiac functions [66]. Hypoxia-inducible factor-1α plays a central role in adaptation to ischemic injury by regulating angiogenesis and glucose metabolism [67]. Dimethyloxalyglycine (DMOG) is a propyl hydroxylase inhibitor that stabilizes hypoxia-inducible factor-1α, thus leading to the accumulation and enhancement of activity. Stem cell preconditioning with DMOG resulted in the better survival of transplanted cells and stimulation of angiogenesis [68,69]. Trimetazidine (TMZ) is another potent anti-ischemic agent, which reduces fatty-acid oxidation, promotes glucose oxidation, and hence lowers the oxygen demand for ATP production [70]. It was reported that the pretreatment of MSCs with TMZ upregulated pAkt and Bcl-2 expression and resulted in a significant improvement of cardiac function and decreased levels of fibrosis in a rat model of MI [71,72].

Statins are a group of lipid-lowering drugs recognized to decrease inflammation and oxidative stress and support endothelial function [73]. Stem cell preconditioning with Atorvastatin is reported to increase the survival of transplanted cells and increase the beneficial effects of stem cell therapy for cardiac function recovery [74] via the RhoA/ROCK/ERK pathway inhibition [73]. Atorvastatin pretreatment has also been shown to upregulate CXCR4, a specific receptor for stromal cell-derived factor-1, which enables cell recruitment to an infarction site. The migration of MSCs is improved with atorvastatin preconditioning both in vitro and in vivo [75]. Rosuvastatin enhanced the proliferation and survival of the engrafted ADSCs. Furthermore, the combination of ADSCs and rosuvastatin decreased fibrosis, inhibited cardiomyocyte apoptosis, and preserved cardiac function [76]. Overall, the use of pharmacological and biological substances benefits stem cell therapy by increasing survival, promoting angiogenesis and differentiation, and leading to greater heart function recovery.

## 3. Genetically Modified Stem Cell for MI Treatment

### 3.1. Genetic Modification

The genetic modification of stem cells to overproduce specific GF, anti-apoptotic proteins, or pro-survival genes is another way to increase transplanted cell survival and engraftment [77]. One study described the transduction of the IL-7 gene into MSCs and their subsequent transplantation into a rat model of MI. As a result, compared to standard MSCs, cells transduced with the IL-7 gene significantly reduced their infarct size and maintained the thickness of the left ventricular wall [78]. Similarly, the transplantation of MSCs overexpressing another cytokine, namely, IL-10, also improved their efficiency [79]. In particular, rats that received the IL-10 overexpressing MSCs had significantly reduced infarct size and greatly improved cardiac function compared to MSCs without IL-10 overexpression. Moreover, the modified MSCs exhibited much more pronounced anti-inflammatory effects compared to IL-10 treatment alone. IL-33 is another cytokine that was utilized for the stem cell therapy of MI. Chen and colleagues discovered that BMSCs overexpressing IL-33 experienced better survival and retention, as well as preserved myocardial function to a greater extent, in comparison to non-modified BMSCs in post-MI rats [80]. Importantly, IL-33-BMSCs caused the M2 polarization of macrophages and reduced the production of inflammatory cytokines IL-6, TNFα, and IL-1β in the border zone of the infarcted hearts. In another study, hypoxia preconditioned MSCs were genetically modified to express growth differentiation factor 11, which is a TGF-β family protein. MI-induced mice injected with recombinant cells demonstrated increased heart function recovery, an increase in vascular density, and a decrease in scar size. Growth differentiation factor 11 was shown to promote cell survival through acting on the Smad2/3/YME1L/OPA1 pathway [81].

The efficiency of MSCs overexpressing HGF was evaluated in a murine model of MI [82]. The HGF-MSCs survived better and exhibited greater paracrine activity by producing higher quantities of HGF, EGF, bFGF, and VEGF compared to conventional MSCs. Moreover, the transplantation of HGF-MSCs resulted in increased cardiomyocyte survival and proliferation, as well as improved cardiac function and angiogenesis. IGF-1 is another GF that was used to enhance the efficiency of stem cell therapy of MI. Lin and colleagues transplanted BMSCs overexpressing IGF-1 to rats with acute MI [83]. IGF-1 overexpressing BMSCs had higher survival and proliferation, protected cardiomyocytes from apoptosis, and reduced the infarct size. The authors reported that the beneficial effects of IGF-1 on BMSCs were mediated to some extent via the AKT/secreted frizzled-related protein 2 (SFRP2) pathway with SFRP2 being an agonist rather than an antagonist in the Wnt/β-catenin pathway.

The clinical use and efficiency of autologous stem cells is currently limited because most MI patients are elderly and the stem cells obtained from these patients are mostly aged [84,85]. The application of the strategy described in this section can be utilized to rejuvenate aged stem cells. In the study by Zhang and colleagues, aged human BMSCs were transfected to overexpress macrophage migration inhibitory factor (MMIF) [86]. MMIF is known to promote cell proliferation and survival, as well as prevent cellular senescence [87,88]. The authors demonstrated that MMIF-transfected MSCs had a higher growth rate, experienced growth arrest later, and survived significantly better in comparison to non-transfected MSCs. The authors claimed that MMIF mediated its protective effects on the aged cells by activating autophagy. In particular, the MMIF-transfected cells showed an elevated expression of Beclin1 and LC3I/II but reduced the expression of p62: the key proteins involved in the regulation of autophagy [89]. The MMIF-transfected MSCs also showed excellent therapeutic efficiency in a rat model of MI. Firstly, they had higher survival rates than aged MSCs without MMIF treatment. Secondly, they inhibited fibrosis and significantly reduced the infarct size four weeks following MI. Lastly, the transfected MSCs mediated angiogenesis, which was evidenced by greater alpha smooth muscle actin density and increased CD31 expression in the infarction region, indicating higher capillary density in the test group compared to the controls.

Stem cells can also be genetically modified to overexpress anti-apoptotic and cytoprotective proteins. For instance, the treatment of a rat heart infarction site with MSCs that overexpressed Lipocalin 2, a cytoprotective factor, enhanced the healing process, reduced fibrosis, and increased cell retention [90]. In another study, the cardioprotective effects of protein kinase C ε (PKCε) were evaluated [91]. PKCε-overexpressed MSCs had improved survival, retention, and differentiation in vitro and in vivo. Furthermore, they enhanced cardiac function, reduced remodeling and infarct size, increased the production of several important paracrine factors, and mediated angiogenesis to a greater extent than non-modified MSCs. Chen and colleagues assessed the efficiency of endothelial nitric oxide synthase (eNOS) overexpressing bone marrow-derived MSCs [92]. The authors reported that eNOS-MSCs survived better, reduced infarct size, improved myocardial function, and promoted angiogenesis more effectively than MSCs without eNOS overexpression. To summarize, various genetic manipulations were performed on stem cells for MI treatment purposes. Genetic modification causing stem cells to overexpress crucial factors, proteins, and peptides, alone or in combination with preconditioning, demonstrated positive effects on heart function. Thus, genetically modified stem cells provide multiple opportunities for clinical research in the field.

### 3.2. Overexpression or Inhibition of Specific MicroRNAs

Specific microRNAs (miRNAs) are upregulated in response to ischemia [93]. MiRNAs are noncoding RNAs that regulate post-transcriptional gene expression. The overexpression or inhibition of specific miRNAs was researched in an attempt to influence pro-survival/apoptotic genes and promote cardiac regeneration following MI. MiRNAs are shown to be involved in cardiomyocyte survival/apoptosis, proliferation, post-ischemic angiogenesis, and fibroblast differentiation into cardiomyocytes [93]. Given the effectiveness of miRNAs in MI therapy, stem cells have also been combined with miRNAs aimed to achieve better regenerative effects. BMSCs overexpressing miRNA-1a have been shown to facilitate cardiomyocyte differentiation in vitro [94]. The transfection of MSCs with a recombinant lentiviral vector to overexpress miRNA-1 resulted in an increased survival of transplanted cells, promoted cardiomyocyte differentiation, and improved cardiac function in a mouse MI model [95]. The overexpression of miR-19a/19b in MSCs was shown to significantly decrease cell apoptosis under hypoxic conditions in vitro. In vivo results suggest the reduction of inflammatory cell infiltration and significant improvement of cell survival in miR-19a/19b–MSCs, possibly through the inhibition of Bim and phosphatase and tensin homolog (PTEN) apoptotic genes [96]. In contrast, the inhibition of miR-15a/15b expression in MSCs was reported to aid stem cell survival and enhance cardiac function recovery in a mouse MI model via the VEGFR-2/PI3K/Akt pathway [97].

Angiogenesis is another important mechanism for MI therapy; the transplantation of MSCs transfected with miRNA-126 can improve angiogenesis and cardiac function in the infarcted hearts, which may be due to stimulation of the AKT/ERK-related pathway [98]. Another possible mechanism of miRNA-126 to increase the survival of MSCs is by the secretion of angiogenic factors and activation of Notch ligand Delta-like-4 [99]. Hypoxia-responsive miRNA-377 directly targets VEGF in MSCs, and the knockdown of endogenous miRNA-377 promotes MSC-induced angiogenesis in the infarcted myocardium [100]. Moreover, miRNA-210 and sphingomyelinase 2 (nSMase2) expressions are significantly higher in HP-MSCs compared to normoxic MSCs. The inhibition of nSMase2 in HP-MSCs results in the decreased expression of miRNA-210 and abolition of the positive effect of stem cell treatment [101]. Thus, miRNAs show potential to ameliorate the effects of stem cell therapy by facilitating stem cell survival, angiogenesis, and cardiomyocyte differentiation and reducing inflammation.

### 3.3. Cell Reprogramming and Stem Cell Differentiation

Factors such as high oxidative stress in the inflamed area, cardiac tissue remodeling, and fibrotic scarring are directly associated with a reduced number of cardiomyocytes in the post-MI area [8]. The loss of cardiomyocytes is an irreversible process. Continuous and progressive injury and the inability to fully regenerate infarcted tissue lead to heart failure and death [102]. One approach to replenish the reduced number of cardiomyocytes is a direct reprogramming of cardiac fibroblasts into induced CMs through a viral introduction of lineage core transcription factors such as Gata4, Hand2, Mef2c, and Tbx5 [103]. However, despite using different methods to increase the conversion rate, the efficiency of this technique is still poor, as the low number of cells finally become fully reprogrammed iCMs [104,105].

Transplantation of cardiomyocytes derived from stem cells is another approach. Cardiomyocytes derived from pluripotent stem cells, including ESC-derived cardiomyocytes (ESC-CM) and iPSCs-derived cardiomyocytes (iPSC-CMs), are structurally and functionally identical to the adult cardiomyocytes [16]. Day 20 iPSC-CMs, when transplanted into the mice MI heart, significantly improve cardiac function and manifest high engraftment, proliferation, and progressive maturation [106]. Human-derived ESC-/iPSC-CMs repair left ventricular function more efficiently than non-differentiated stem cells due to their differential paracrine effects [107]. There are three main steps that define stem cell differentiation into cardiomyocytes [108]. In the first step, stem cells are differentiated into cardiac mesoderm via the introduction of Activin A, bone morphogenetic protein 4, FGF2, VEGF, and Wnt/β-catenin factors [109,110]. Then, the cells are exposed to dickkopf homolog 1 (DKK1) or the inhibitors of Wnt/β-catenin and Activin/Nodal to transform them into cardiac progenitors [111,112]. Lastly, the cells are differentiated into cardiomyocytes and induced to proliferate through the regulation of HGF, platelet-derived growth factor, Neuregulin-1, Notch-1, and Wnt/β-catenin [60,112]. In addition, FGF promotes cardiomyogenesis via the activation of phosphoinositide 3-kinase (PI3K), while platelet-derived growth factor modulates cardiomyocytes’ proliferation through the activation of the Akt pathway and inhibition of GSK-3β and cyclin-dependent kinase (CDK) inhibitor p27. Neuregulin-1 induces DNA synthesis via the ErbB4 receptor [113]. Recently, a mass production of human PSC-CMs in chemically defined conditions was described. Uninterrupted chemical control of the WNT pathway at the beginning of differentiation allowed for an effectiveness of the method and led to the production of ventricular-like CMs [114]. An initial feature of differentiated cardiomyocytes is an increase of early (i.e., NKX2.5, myocyte enhancer factor 2C (Mef2c) and GATA4) and mature (i.e., troponin T (cTnT), heavy chain cardiac myosin (MYH6), and connexin 43 (Cx43) cardiac-specific progenitor markers [115].

In previous studies, c-kit+ cells were observed to be overproduced in post-MI heart and believed to facilitate cardiac recovery as well as differentiate into cardiomyocytes. Therefore, c-kit was used as a cardiac progenitor cell marker [116,117]. Recently, several studies revealed that the contribution of c-kit+ cells to the generation of cardiomyocytes is insignificant due to their heterogeneous nature [117,118,119].

**Figure 2:**
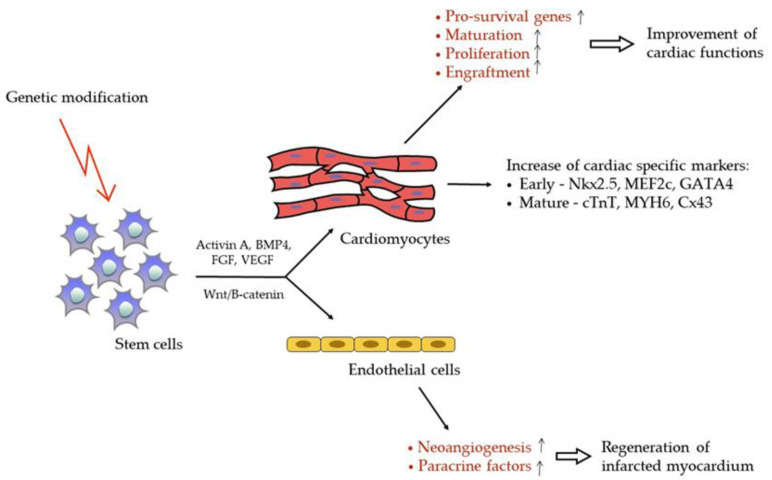
Genetic modification induces stem cells differentiation toward cardiomyocytes and endothelial cells. The process is regulated by Activin A, bone morphogenetic protein 4 (BMP4), basic fibroblast growth factor (FGF2), vascular endothelial growth factor (VEGF), and Wnt/β-catenin factors. Cardiomyocytes derived from stem cells manifest an increased expression of pro-survival genes, progressive maturation, proliferation and high engraftment; those beneficials contribute to a significant improvement of cardiac functions. Endothelial cells promote neoangeogenesis and secrete paracrine factors which help to regenerate infarcted myocardium. Arrows designate an increase in corresponding functions.

One way to induce stem cell differentiation into cardiomyocytes is to genetically modify them (Figure 2). Overexpression of the cardiac troponin I-interacting kinase (TNNI3K) gene enhanced the differentiation of ESCs toward cardiomyocytes as demonstrated by the increased levels of cardiac lineage markers (i.e., α-actinin, MLC2v, MHC6, cx43, cTnl, and cTnT) and transcription factors (i.e., GATA4 and Nkx2.5). The underlying mechanism includes suppression of the ERK pathway for p38 as well as c-Jun-NH(2)-terminal kinase (JNK)-modulated apoptosis and regulation of PKCε/p-PKCε to overproduce cTnT-positive cells [120]. Moreover, the treatment of mouse BMSCs with miR1-2 increased the differentiation of BMSCs into cardiomyocytes through the activation of the Wnt/β-catenin signaling pathway. In addition, the overexpression of miRNA1-2 significantly reduced the apoptosis of BMSCs [121].

In their in vitro pre-differentiation protocol, which combined primary cardiac feeder layers and aggregate formation, Szaraz and colleagues used first-trimester human umbilical cord perivascular cells (FTM HUCPVCs), as they allowed for the better formation of aggregates and demonstrated comparatively low immunogenicity. FTM HUCPVCs showed a significantly enhanced capability to differentiate into cardiomyocytes compared to conventional BMSCs and contributed to an increased production of spontaneously contracting cardiomyocyte-like cells. The increased expression of cardiac-specific marker genes (i.e., myocyte enhancer factor 2C, aSarc) as well as intracellular (i.e., cTnT and MYH6) and cell-surface proteins (i.e., SIRPA, Cx43) were revealed [115]. Furthermore, the preconditioning of hiPSC-CMs with Y-27632 prior to injection into a mouse MI model increased the engraftment rate seven times through the enhancement of transplanted cell survival and regulation of cell adhesion that was promoted by the upregulation of junctional proteins, such as integrins and N-cadherin [122].

Stem cell commitment to cardiomyocytes can also be induced by physical forces. Electrical stimulation (EleS) can significantly enhance the differentiation of hiPSCs toward cardiomyocytes and support their maturation. EleS activates Ca2+/PKC/ERK signaling pathways, which leads to an expression of essential cardiac transcription factors and genes such as GATA4, MEF2, ACTCT1, HAND2, and TBX5. As a result, EleS-preconditioned CMs reduced fibrosis and contributed to better myocardial regeneration in a mouse model of MI [123]. Preconditioning stem cells with dynamic mechanical strain causes cardiomyogenic differentiation. The short-term stimulation of adult human periodontal ligament derived stem cells (PDLSC) through tensile mechanical strain induces cardiomyogenesis, activates cardiac growth factors (i.e., GATA4, MEF2C, Nkx2.5), and promotes the production of sarcomeric actin, cTnT, and nitric oxide. In addition, PDLSCs are a promising source for MI treatment [116].

BMSCs can also be differentiated into endothelial cells, which have been revealed to regenerate post-MI myocardium by promoting neoangeogenesis and secreting paracrine factors (Figure 2) [106]. An enhanced expression of VEGF in vascular endothelial cells following MI contributes to angiogenesis via the increased reactive oxygen species and endoplasmic reticulum stress-mediated autophagy [124]. Moreover, the differentiation of PSCs into endothelial cells is regulated similarly to cardiomyocyte factors, such as bone morphogenetic protein 4, Activin A, VEGF, and bFGF, due to a shared signaling pathway in cardiogenic or hemogenic mesoderm [111]. In general, the number of cardiomyocytes decreased in the infarcted heart and lost their proliferation abilities in vivo. Therefore, in vitro manipulations with stem cells that stimulate their differentiation into other cell types, such as cardiomyocytes, significantly improve cardiac function and regenerate infarcted myocardium.

Human PSC could also be utilized for heart repair; however, there are some issues related to their use. For example, poor survival, low retention of stem cells, immune system rejection, and a lack of tools allowing the long-term tracking of viable transplanted cells appear to be significant limitations of this strategy [125,126]. A recently published large study on a primate MI model revealed that hPSCs do not result in engraftment and remuscularization at 140 days after transplantation, despite the use of multiple immunosuppressive regimens [127]. Moreover, hepatic injury was associated with immunosuppressive therapy. Another study showed that human ESC-derived cardiomyocytes show good potential for heart regeneration but induce ventricular tachyarrhythmia [128]. Nevertheless, some progress was made to address these issues. Particularly, human ESCs in a cocktail of factors targeting potential death pathways showed improved survival [129]. It was demonstrated that one-to-one electrophysiological coupling between the graft and host cardiac cells enhanced the engraftment of human ESC-derived CMs and suppressed ventricular arrhythmia [130]. Moreover, Shiba and colleagues reported reduced immunogenicity in the MHC-matched Cynomolgus monkeys upon the allogenic transplantation of iPSC-CMs [131].

Advancements in the methods of improving the effectiveness of stem cell therapy for MI treatment possess a promising potential for clinical application. Particularly, the growing field of genetic engineering provides a wide range of possibilities to overexpress or inhibit the expression of specific factors, proteins, and genes. However, further extensive studies are needed for the usage of the genetically manipulated stem cells in a clinical setting of MI repair due to the possibility of unexpected consequences. Therefore, at the moment, pre-culturing in a hypoxic environment and/or with growth factors, drugs, and biological agents is preferable. In addition, the preconditioning of stem cells in hypoxia has already shown a positive outcome in preclinical studies on non-human primates, and cells precultured with GFs were also successfully used on various clinical conditions.

## 4. Conclusions

Cardiovascular diseases remain a leading cause of mortality worldwide, and myocardial infarction is considered a major contributor to this cause. In preclinical studies, stem cell-based therapies have shown to be effective in enhancing the function of hearts following myocardial infarction. However, certain limitations of the stem cells, such as low engraftment and poor survival in the infarction site, prevent the implementation of the treatment in clinical practice. Recent advancements in the field of stem cell therapy provide promising results to improve the effectiveness of cell transplantation via different approaches. These include cell preconditioning in a hypoxic environment or with growth factors and the application of chemical substances/drugs or genetic manipulations by overexpressing certain proteins in the cells prior to transplantation into the infarction area. The recent discoveries summarized in this study demonstrate the potential of these various approaches of stem cell function enhancements to translate preclinical myocardial infarction treatment methods to the bedside. However, animal models do not fully replicate all processes that take place in humans following myocardial infarction, and the results of preclinical studies should be carefully interpreted.
